# Recent Advances in Betaine: Extraction, Identification, and Bioactive Functions in Human Health

**DOI:** 10.1002/fsn3.70173

**Published:** 2025-04-17

**Authors:** Donglan Luo, Xiaogang Wang, Sen Cao, Liangjie Ba, Jianye Chen

**Affiliations:** ^1^ Guiyang University Guiyang Guizhou China; ^2^ State Key Laboratory for Conservation and Utilization of Subtropical Agro‐Bioresources/Guangdong Provincial Key Laboratory of Postharvest Science of Fruits and Vegetables/Engineering Research Center of Southern Horticultural Products Preservation, Ministry of Education, College of Horticulture South China Agricultural University Guangzhou Guangdong China

**Keywords:** applications, betaine, bioactivity, health effects, synthesis

## Abstract

Betaine plays a crucial role in regulating the physiological metabolism of plant fruits and exhibits specific biological activities across animals, plants, and microorganisms. This review highlights that betaine can serve as a nutritional supplement with antioxidant, anti‐inflammatory, and antifungal properties. Specifically, through its antioxidant activity, betaine enhances liver detoxification, reduces cell apoptosis, potentially mitigates the progression of liver fibrosis, and exerts beneficial effects on radiation‐induced liver injury. A growing body of research utilizing animal models has indicated that betaine, due to its unique bioactivity, may be integral to both human health and industrial progress. However, there remains a notable scarcity of reviews focusing on the biological synthesis of betaine. This paper presents a comprehensive and up‐to‐date account of the natural sources and concentrations of betaine, encompassing its biosynthetic pathways, bioactive functions, and potential mechanisms underlying its effects on human health. Special emphasis is placed on the biological effects of betaine in animal therapy as well as its mechanisms influencing human health. Furthermore, recommendations are offered for optimizing the utilization of betaine.

## Graphical abstract

Betaine exhibits a range of biological activities, including antioxidant properties and the ability to enhance the preservation of fruits and vegetables post‐harvest. Betaine contributes positively to human health and is associated with various beneficial effects on well‐being. There is potential for betaine to be utilized as a nutritional supplement in food applications.

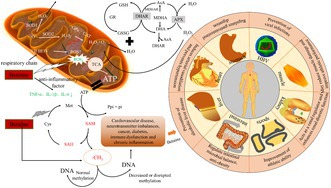

## 1 Introduction

Betaine (GB) is a water‐soluble nitrogenous compound and a three‐methyl derivative of glycine, found in plants, animals, and microorganisms (Dobrijević et al. 2023). Beet pigments include purplish‐red betaines and yellow‐orange betaines.

Betaine, an endogenous plant compound, promotes health in animals upon ingestion and may benefit human health. It is produced via choline metabolism or ingested from food or supplements (Willingham et al. 2020). Its bioavailability is similar regardless of the sources. Betaine inhibits oxidative stress, scavenges ROS, and mitigates NaAsO‐induced kidney injury in mice (Norouzzadeh et al. 2024). It enhances athletic performance by improving endurance, reducing fatigue, and increasing muscle strength (Cholewa et al. 2019). Betaine also protects the liver and prevents injury due to its anti‐inflammatory and antioxidant properties (Heidari et al. 2018; Arumugam et al. 2021). In mice, betaine reduces pain responses in a dose‐dependent manner. Combined with naloxone or flumazenil, it decreases licking and biting times and improves injury resistance. Betaine lowers MDA levels while raising SOD and GSH‐Px levels in formalin‐treated mice without adverse effects (Hassanpour et al. 2020). In RAW 264.7 cells, betaine suppresses pro‐inflammatory markers (IL‐6, IL‐1β, iNOS, COX‐2) and NOX‐2 mRNA levels, counteracts hydrogen peroxide‐induced ROS, and demonstrates free radical scavenging potential (Ghahramani et al. 2016). Astaxanthin, in contrast, elevates ROS production beyond hydrogen levels (Fernando et al. 2023). Betaine's diverse bioactivities promote health and prevent disease, attracting significant research interest (Henarejos‐Escudero et al. 2022).

Betaine stabilizes cell membranes, protects proteins, and enhances resistance to abiotic stresses, improving the postharvest quality of fruits and vegetables. In a recent study, betaine mitigated weight loss and firmness degradation in ‘Huangguoggan’ during postharvest storage at ambient temperature. It enhanced antioxidant enzyme activity, maintained phenolic and flavonoid levels, and reduced accumulation of H_2_O_2_. The application of betaine preserved the postharvest storage quality and extended the shelf life of “Huangguoggan” (Zheng et al. 2023). Furthermore, a polycaprolactam‐betaine solution exhibited bacteriostatic properties against eight ATCC strains as well as 21 multidrug‐resistant (MDR) clinical strains including *Staphylococcus aureus* , *Enterococcus faecalis* , *Escherichia coli* , *Enterobacteriaceae* species, *Enterococcus anguillaris*, *Klebsiella pneumoniae* , *Fusobacterium baumannii*, and *Pseudomonas aeruginosa* . The minimum inhibitory concentrations (MICs) for polycaprolactam‐betaine ranged from 0.5 to 8 mg/L when assessed through dilution‐neutralization methods. Similarly, polyhexamide‐betaine displayed MIC values within this range while demonstrating zero inoculum clearance across all eight tested strains using the same method. Notably, polyhexamide‐betaine exhibited fungal activity at concentrations significantly lower than those required by commercial alternatives (López‐Rojas et al. 2017). Additionally, the antioxidant and anti‐aging effects of betaine were further evaluated for their in vivo activity; results indicated that betaine extended the lifespan of *Cryptococcus hidradii* by approximately 7%.

Overall, prior reviews of betaine's health benefits and applications have shown consistency but lack comprehensive integration of evidence its synthesis and diverse sources. Further investigation is essential to elucidate the mechanisms of action and efficacy of betaine within the human organism. Although its health benefits are well‐documented (Cheok et al. 2020), research on its metabolic regulation and protective effects in biological systems remains limited. Additionally, clinical studies on betaine are notably limited. Therefore, it is essential to gain further molecular mechanistic insights into characterizing betaine's actions and to more clearly identify its molecular targets (Madadi et al. 2020).

This review aims to compile evidence on betaine's benefits, analyze its sources, synthesis, biological activities, and functions in animal models, and identify trends and mechanisms of its health‐promoting effects. To provide some reference for the extraction as well as application of betaine.

## 2 Betaine in Nature

Betaine is predominantly distributed in significant quantities across diverse botanical species, including *Beta vulgaris* (beet), *Cactaceae*, cereal crops, and *Amaranthus* spp. (Table 1). The majority of betaine is sourced from beet, with its total antioxidant capacity attributed to the synergistic effects of betaine derivatives and polyphenolic constituents (Preczenhak et al. 2019).

**TABLE 1:** Content of betaine.

Source	Betaine content (μg/g)	Reference
Bran of wheat grain	2300–7200	Filipčev et al. 2015
Germ	3414	
Buckwheat	3930	Ross et al. 2014
Buckwheat uncooked pasta	390	
Wheat	747–2899	Patterson et al. 2018
Rice	0–10	
Maize	0–10	
Alfalfa Sprouts	240	Bruce et al. 2010
Brussel Sprouts	34	
Grapefruit	190	
Lemon	120	
Mushroom	15	
Orange	510	Dobrijević et al. 2023
Raw amaranth seeds	7420	
Silverbeet	50	
Monkfish	40	
Mussel	26	
Tea	< 10	
Farina tritici	270–1110	
Oat	200–1000	
Pitaya	2600	Rodríguez‐Félix et al. 2023
Beetroot	3980	Kumar et al. 2023
*N. benthamiana* leaves	110 to 1066	Pramanik et al. 2024
Peach	150–200	Shan et al. 2016
*Beta vulgaris* (golden)	4330–5070	Rivoira et al. 2017
*Beta vulgaris* (red)	1960–3880	

Basellaceae represents a promising source of natural pigments, including betaines (Sutor‐Świeży et al. 2022). Additionally, *Ulluco* ( *Ullucus tuberosus* ), which contains betaine, is an important Andean crop known for its efficacy in treating burns and preventing scarring (Campos et al. 2018).

Dragon fruit has attracted extensive research owing to its high betaine content, which distinguishes it from other common fruits. It is also a source of vitamin C and antioxidants, including phenolic compounds and betaine (Angonese et al. 2021). Dragon fruit can be cultivated in a variety of dryland environments, potentially contributing to the sustainable development of the inhabitants in these regions (Ramírez‐Rodríguez et al. 2020). Furthermore, the stems and flowers of *Amaranthus* have been found to produce astaxanthin and betaine (Spórna‐Kucab et al. 2022). This plant is utilized as an anthelmintic and antiseptic in numerous countries, and it is also employed in the treatment of burns, headaches, gastric disorders, hepatic issues, intestinal ailments, arthritis, diabetes mellitus, and other diseases. The petal extract of amaranths tinctorius underwent high‐performance liquid chromatography (HPLC) analysis, which revealed two major yellow pigments. It has been reported that cereals contain a certain amount of betaine (Corol et al. 2012). As noted by Corol et al. (2012), the betaine content within grains is influenced by various factors, including genotypic variations and environmental conditions such as geography and climate change interactions with genotype. Amaranth is classified as a pseudocereal and is recognized as one of the richest sources of betaine (Table 1).

## 3 Synthesis and Pproduction Pathways of Betaine

Betaines are natural N‐heterocyclic pigments that function as water‐soluble plant pigments. The synthesis of betaines primarily occurs through enzyme‐catalyzed reactions, while animals acquire the intermediate product betaine aldehyde via a two‐step pathway. In the initial step, choline is catalyzed by the enzyme choline dehydrogenase (CDH), followed by the formation of betaine through the action of betaine aldehyde dehydrogenase (BADH), also referred to as betaine aldehyde dehydrogenase (Glabonjat et al. 2018).

**FIGURE 1:**
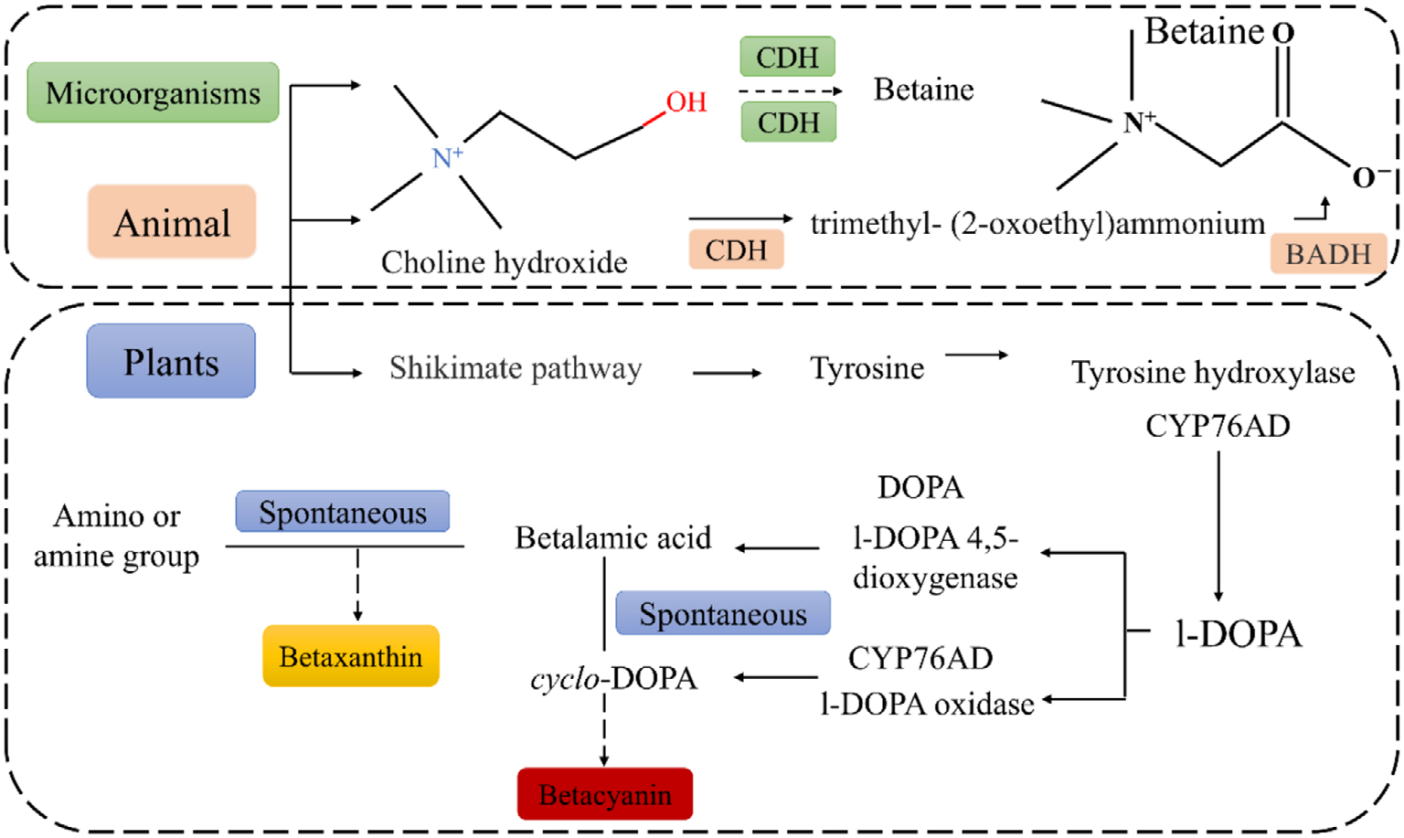
Betaine biology and chemical synthesis.

Betaine is synthesized from tyrosine, which originates from the shikimate pathway and serves as a precursor for betaine synthesis. Tyrosine undergoes hydroxylation to be converted into levodopa, facilitated by cytochrome P450 enzymes. Subsequently, L‐3,4‐dihydroxyphenylalanine (L‐DOPA) experiences ring cleavage mediated by L‐DOPA4,5‐dioxygenase (DODA) in a ring‐opening oxidation reaction, resulting in the intermediate compound 4,5‐seco‐DOPA. This intermediate then undergoes spontaneous intramolecular condensation to yield *β*‐thalamic acid. Further oxidation leads to the production of levodopa; this step is likewise catalyzed by cytochrome P450 enzymes (Amorim et al. 2023). Of particular significance are cyclo‐DOPA and malonic acid, which act as essential intermediates in the synthesis of betacyanin and betaxanthin. Spontaneous condensation reactions between malonic acid and amino acids or amines lead to the formation of betaines. In most plants, the arylate precursor is catalyzed by arylate dehydrogenase (ADH;3) (Carreón‐Hidalgo et al. 2022). Plants utilize choline monooxygenase (CMO) for the generation of betaine aldehyde during choline metabolism. Conversely, microorganisms typically catalyze this reaction twice through choline dehydrogenase (CDH) to produce betaine. However, certain bacteria and fungi possess the ability to directly oxidize choline using choline oxidase (COD) in an oxygen‐rich environment. This process entails the direct oxidation of choline, with oxygen serving as the primary electron acceptor, resulting in both the production of betaine and the formation of betaine aldehyde, as illustrated in Figure 1.

## 4 Extraction Process of Betaine

Given that betaine is thermally unstable, several critical factors must be taken into account during the extraction of this compound (Rocha et al. 2022). These factors encompass extraction time, extraction temperature, solution pH, solvent type, enzymatic presence or absence, and the specific extraction method employed. Additionally, advanced techniques such as microwave‐assisted extraction or electric pulse treatment may also be integrated into this process (Zin et al. 2021). It is evident that these various extraction methods will inevitably influence betaine levels. Therefore, identifying an optimal extraction process is essential for enhancing both the rate of betaine recovery and its stability. The enzyme‐based extraction method primarily facilitates betaine recovery by disrupting cell walls. Optimal conditions for this approach have been established as follows: an enzyme concentration of 25 U g^−1^, a temperature of 25°C, and a duration of 4 h. Under these conditions, resulting concentrations were measured at 14.7 mg betacyanins 100 mL^−1^ and 11.4 mg betaxanthins 100 mL^−1^ (Lombardelli et al. 2021). This method has proven to be highly effective in improving both the rate of extraction and ensuring stability; however, it remains limited by temperature constraints. Microwave‐assisted extraction represents a novel and environmentally friendly technique for recovering betaine. The optimal parameters identified for this method include a temperature setting of 35°C, a sample mass of 20 g, and a processing time lasting 8 min. Utilizing these conditions yielded a betaine content quantified at 9 mg L^−1^ (Thirugnanasambandham and Sivakumar 2017). Supercritical fluid extraction (SFE) has been shown to produce a higher yield of extracted solution compared to conventional extraction methods. The peel and pulp extracts obtained through SFE using a 10% ethanol/water (v/v) mixture as a co‐solvent at 25 MPa pressure exhibited total betaine levels of 24.58 mg 100 mL^−1^ and 91.27 mg 100 mL^−1^, respectively. The optimal solvent extraction process resulted in relatively high concentrations of total betaines in the peel and pulp extracts, measuring 28.44 mg 100 mL^−1^ and 120.28 mg 100 mL^−1^, respectively.

The predominant betaines identified in the dragon fruit peel and pulp extracts included betaines, isobetaines, chlorophylls, butyryl betaines, isochlorophylls, and isobutyryl betaines. Furthermore, employing a higher ratio of ethanol to water (E/W) during the extraction process led to increased antioxidant activity in the pulp extracts compared to those from the peel (Fathordoobady et al. 2016). The influence of operational variables—namely temperature, time, and the ethanol‐to‐water ratio—on the levels of extracted betaines was evaluated using a Box–Behnken experimental design. The findings indicated that the concentrations of extracted betaines (0.11–4.24 mg g^−1^) surpassed those of beet xanthophylls (0.02–2.89 mg g^−1^). Among these variables, the concentration of ethanol in the extraction solvent exhibited the most pronounced effect on extraction efficiency. While elevated temperatures adversely affected betaxanthin extraction, they did not significantly impact betacyanin removal. A comparison with conventional extraction methods revealed that optimal conditions for extracting both betacyanins and betaxanthins were achieved at temperatures of 52°C and 37°C, respectively, with an extraction duration of 90 min utilizing a 25% aqueous ethanol solution as a solvent. Beet pulp is subjected to water extraction, and then the solution containing betaine is purified by methods such as ion exchange chromatography. Finally, betaine is concentrated and converted into a crystalline or liquid form for sale as an industrial raw material. Sugar factories that have the appropriate installations can carry out these processes themselves or cooperate with companies specializing in the recovery of betaine from by‐products. In this way, they optimize the use of the raw material and increase the economic efficiency of their plants (da Righi Pessoa Silva et al. 2018).

In another study, ultrasound‐assisted extraction emerged as a more effective technique for efficiently isolating betaine from dried fruit pulp. The total betaine demonstrated favorable storage stability and antioxidant capacity for up to 4 weeks post‐extraction from dried beet waste (Fernando et al. 2021). However, it is important to note that the extraction process remains limited by factors such as temperature and time. Despite the multitude of methods available for extracting betaine, each approach presents distinct limitations and high costs that pose significant challenges for industrial‐scale production. Consequently, there is a clear necessity to further explore cost‐effective extraction processes characterized by high yields to facilitate their integration into industrial production systems while positively contributing to economic development.

## 5 Betaine Bioactivity

**FIGURE 2:**
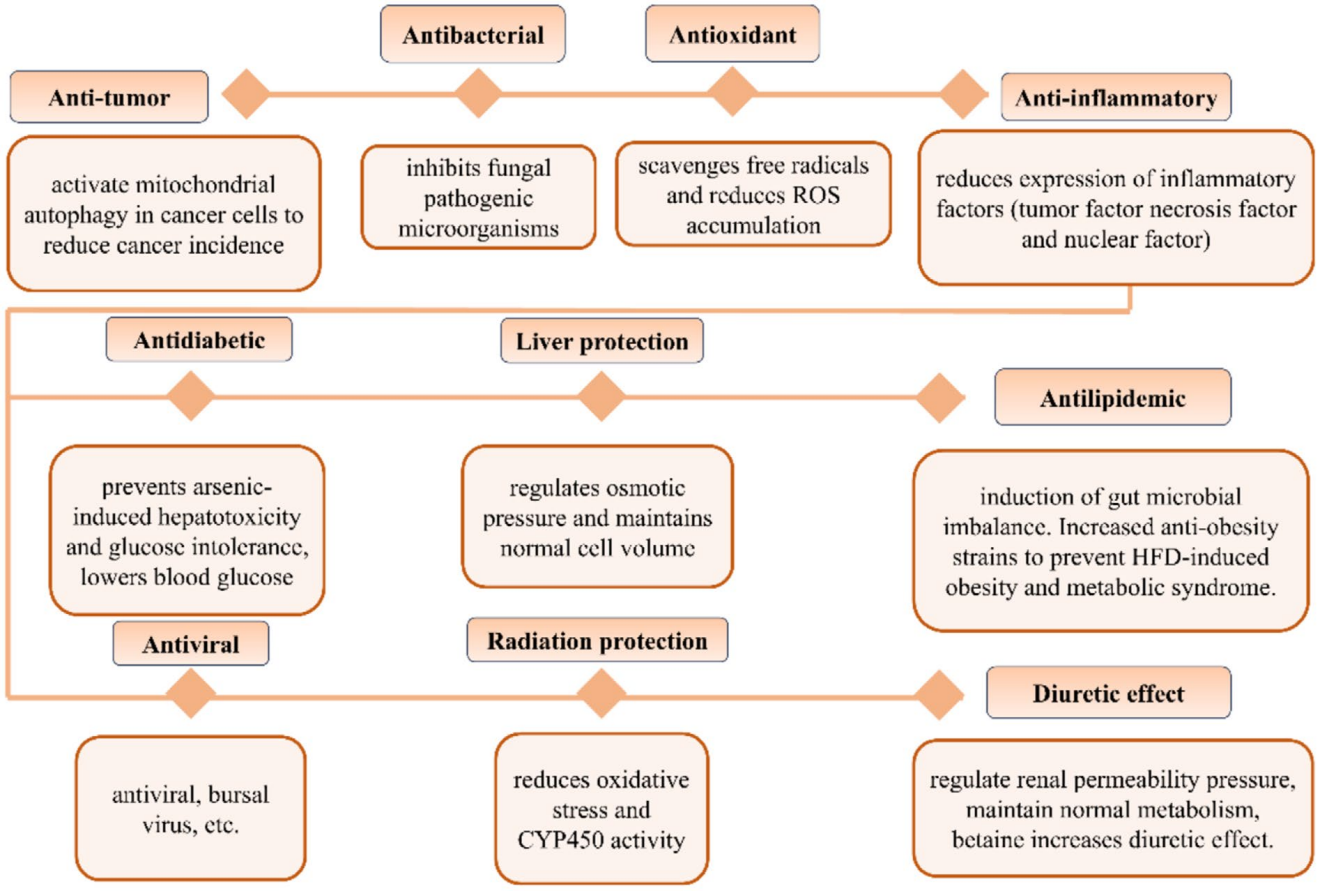
Biological activity of betaine.

The nutrient betaine has attracted considerable research interest due to its potential health benefits. Emerging evidence indicates that bioactive compounds may enhance human health with minimal adverse effects. A substantial body of literature suggests that betaine is a promising candidate for the development of novel functional foods, primarily because it serves as a rich reservoir of biologically active phytochemicals (Figure 2).

### 5.1 Antioxidant Activity

It has been reported that dragon fruit peel contains high levels of phenolic compounds, and the peel extract demonstrated potent antioxidant activity based on the assessment of its free radical scavenging capacity using the DPPH and ABTS assays (2,2′‐azino‐bis) (3‐ethyl‐benzothiazoline‐6‐sulfonic acid) (Wu et al. 2020). Similarly, another study demonstrated that the methanolic extract of dragon fruit, as determined by the DPPH method, exhibited a robust antioxidant capacity, which could be attributed to the high content of phenolic compounds, with a value of 48.15 mg GAE 100 g^−1^ (Som et al. 2019). Dragon fruit contains a relatively low concentration of non‐betaine phenolic compounds, yet its antioxidant capacity is primarily attributed to the presence of betaine. Some studies have demonstrated that at pH > 4, the free radical scavenging capacity of betaine is nearly twice that of certain anthocyanins. Harahap and Amelia (Harahap and Amelia 2019) demonstrated that the intake of betaine reduces the increase in ROS due to strenuous exercise, decreases oxidative stress, repairs cellular function, and delays fatigue. In conclusion, the antioxidant activity of betaine derived from dragon fruit is noteworthy, and it holds promise as a natural or synthetic antioxidant for use in various food products, such as fruits, vegetables, fish, meat, and other foods. Future research could further explore the application of extracted betaine by transitioning from animal modeling studies to clinical trials, thereby providing deeper insights into its mechanism of action.

### 5.2 Anti‐Tumor Activity

Cancer is a significant contributor to increased mortality and a major cause of numerous complications. These complications are challenging to treat and often expensive, representing a significant threat to human health. Prior to 1996, Govind J. Kapadia et al. initiated studies on the antitumor activity of betaine derived from beetroot (Kapadia et al. 1996). The findings demonstrated that the administration of betaine via the oral route to ICR mice that had been pretreated with the tumor promoter 12‐O‐tetradecanoylphorbol‐13acetate (TPA) led to the inhibition of skin and lung tumorigenesis. Notably, the reduction rate in lung tumors reached 60%. This is the inaugural report to elucidate the anti‐tumor activity of betaine in beet using an animal model. Subsequently, Kapadia et al. (2003) three distinct murine tumor models were constructed in experiments, including those induced by 7,12‐dimethylbenz(a)anthracene (DMBA), (±)‐(E)‐4‐methyl‐2‐[(E)‐hydroxyamino]‐5‐nitro‐6‐methoxy‐3‐hexanamide (NOR‐1), and TPA, as well as those induced by N‐nitrosodiethylamine (DEN) and phenobarbital. The results demonstrated that betaine exhibited a notable capacity to impede tumorigenicity in the presence of chemical carcinogens (Kapadia et al. 2003). In HepG2 cells, betaine has been demonstrated to induce nuclear translocation of Nrf2, which in turn results in cell death (Krajka‐Kuźniak et al. 2013). It is noteworthy that betaine does not exert any adverse effects on normal cells or physiological metabolism. Moreover, betaine has been demonstrated to possess notable cytotoxic properties against cancer cells (Nowacki et al. 2015). Furthermore, betaine treatment inhibited the proliferation of papillomas in rats administered N‐nitrosomethylbenzylamine (NMBA) and the development of esophageal carcinomas in these treated rats (Lechner et al. 2010). Betaine has been demonstrated to induce apoptosis in cancer cells and to activate mitochondrial autophagy in SW480 and SW620 colon cancer cells. Betaine has been demonstrated to inhibit cell viability in a temporal and dosage‐dependent manner, while also promoting cell cycle arrest in the G2/M phase and the accumulation of cyclin A and cyclin B cells (D'Onofrio et al. 2021). In summary, betaine is expected to be used in the treatment of a variety of tumors.

### 5.3 Anti‐Iinflammatory Activity

**FIGURE 3:**
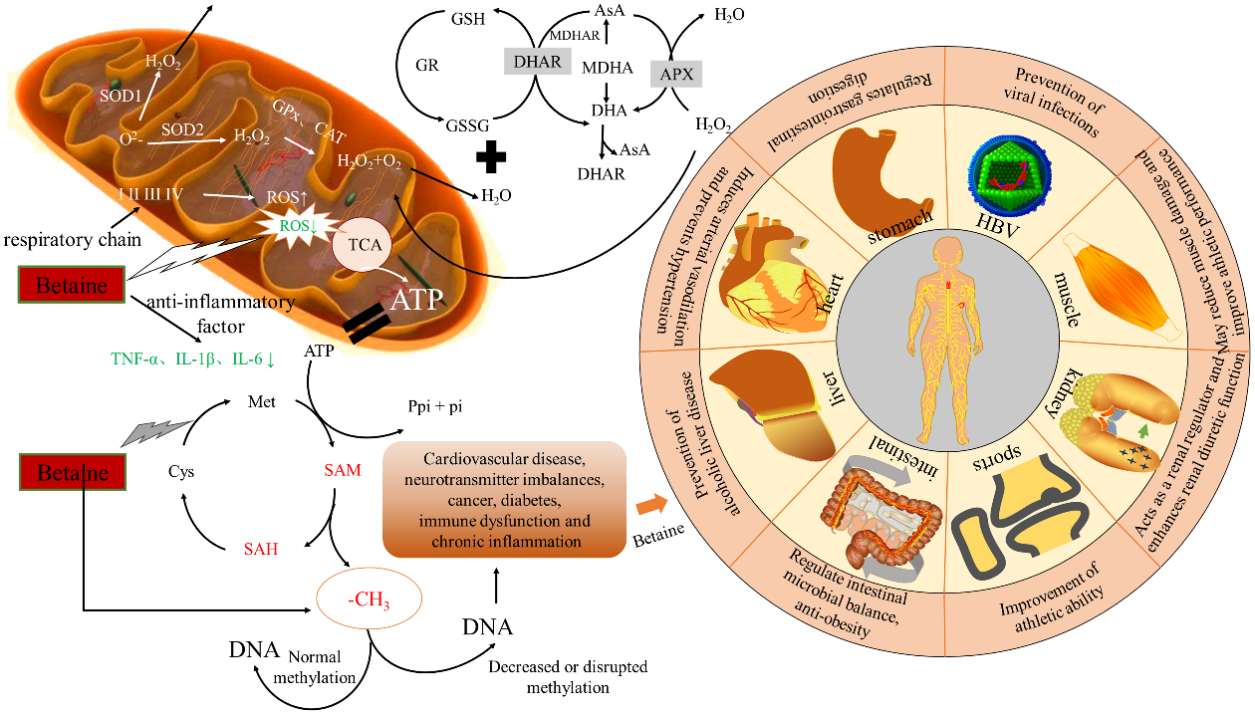
Antioxidant mechanisms of betaine on post‐harvest fruits and vegetables, effects on human pathogenesis and health.

In addition to its antioxidant effects, betaine has been demonstrated to possess anti‐inflammatory properties. Inflammation is an immune response for defense and healing, but chronic inflammation can cause disease. ROS are by‐products of metabolism. Antioxidants like catalase, SOD, melatonin, and GSH help remove ROS and free radicals. However, excessive ROS can lead to inflammation and other pathological conditions (Freitas et al. 2016). Betaine regulates inflammation‐related genes via the NF‐κB pathway, including TNF‐α, IL‐1β, and IL‐23. NF‐κB is a key mediator of inflammation in experimental steatohepatitis, and betaine inhibits its activity and downstream genes (Lee et al. 2013). Betaine treatment suppressed NF‐κB activity and downregulated the expression of multiple pro‐inflammatory genes in aged kidneys, including tumor necrosis factor‐α (TNF‐α), vascular cell adhesion molecule‐1 (VCAM‐1), intercellular adhesion molecule‐1 (ICAM‐1), inducible nitric oxide synthase (iNOS), and cyclooxygenase‐2 (COX‐2). These findings indicate that betaine mitigates inflammation by inhibiting the NF‐κB signaling pathway (Zhao et al. 2018). In a study conducted by Shariati et al. (2024), 40 male mice were randomly divided into four groups: (I) control; (II) GB (500 mg/kg); (III) NaAsO_2_ (50 ppm); and (IV) NaAsO_2_ + GB. Following an eight‐week period during which the animals ingested NaAsO_2_, betaine was administered to those that had been given NaAsO_2_ 2 weeks prior. Following the mice's execution, the corresponding parameters were measured and analyzed. The results demonstrated that GB administration led to a reduction in alanine aminotransferase, creatine kinase MB, thiobarbituric acid‐responsive substance levels, tumor necrosis factor‐α content, and nuclear factor *κ*B expression levels. Furthermore, betaine was found to enhance the activity levels of cardiac total thiols and catalase, superoxide dismutase, glutathione peroxidase, as well as the expression of nuclear factor erythroid‐2. GB mitigated the detrimental effects associated with the imbalance between oxidative and antioxidant pathways, along with the adverse consequences resulting from histopathological alterations observed in NaAsO_2_‐intoxicated mice. This resulted in a reduction of oxidative stress‐induced damage and inflammation (Shariati et al. 2024). Microglia are among the most significant cells involved in neuroinflammation and are instrumental in the production of free radicals. It has been demonstrated that the production of NO is not mediated by iNOS proteins in microglia, which induce oxidative stress and ROS formation. In a study, betanin was observed to inhibit the production of inflammatory mediators, including TNF‐α, IL‐1β, IL‐6, and free radicals, as well as modulate mitochondrial membrane potential (MMP), lysosomal membrane permeability (LMP), and ATP in microglia at a concentration of 500 μM (Figure 3). Moreover, the findings indicated that the active sites of TNF‐α, IL‐6, iNOS, and NF‐κB were inhibited. This resulted in a reduction in LPS‐induced microglia activation, as evidenced by the inhibition of TNF‐α, IL‐6, iNOS, and NF‐κB active sites. The anti‐inflammatory effect of betaine on activated microglia was demonstrated by Ahmadi et al. (2020). The administration of betaine was observed to significantly reduce the rate of colon tumor formation induced by azoxymethane (AOM) and dextran sulfate sodium salt (DSS) in mice. Additionally, it was noted to inhibit ROS production and GSSG concentration in the colonic mucosa. Betaine was observed to inhibit the NF‐κB pathway and the production of pro‐inflammatory cytokines in RAW 264.7 murine macrophages in response to LPS stimulation (Figure 3). The studies indicate that betaine may be a promising candidate for the development of anti‐inflammatory drugs.

### 5.4 Antimicrobial Activity

Betaine has antimicrobial activity, with minimum inhibitory concentration (MIC) values ranging from 3.9 to 31.2 μg mL^−1^, and antifungal activity, with MIC values spanning from 7.8 to 62.4 μg mL^−1^. Furthermore, certain betaines demonstrated significant antimicrobial biofilm activity, exhibiting IC50 values between 2.1 and 25.3 μg mL^−1^ against fungal strains (Yasa et al. 2017). The antimicrobial activity and preservative effect of dragon fruit extract are attributed to the presence of betaine and phenolic compounds. The betaine‐gelatin film demonstrated efficacy in preventing microbial infections of cod during packaging, exhibiting a notable slowing of fungal growth. This was observed in aerobic, anaerobic, choleretic, and proteolytic hydrolyzing bacteria, as well as in aerobic, anaerobic, and proteolytic hydrolyzing fungi in the dragon fruit‐PLA film (Castro‐Enríquez et al. 2023). It is noteworthy that betaines containing different functional groups also demonstrate antimicrobial activity. Betaine demonstrated notable inhibitory efficacy against gram‐negative bacillus, s. aureus, and e. coli, with minimum inhibitory concentrations (MICs) of 61 and 120 μM against *s. aureus* and *e. coli*, respectively. However, it was discovered that the inhibitory capacity of betaine was closely associated with the length of the alkyl chain, which determines the minimum inhibitory concentration and the size of the zone of inhibition for the growth of the compound. Furthermore, this compound exhibited a notable antimicrobial activity (Wieczorek et al. 2017) (Figure 2). It is anticipated that betaine will be employed extensively in the future for the prevention of microbial infections in food.

**FIGURE 4:**
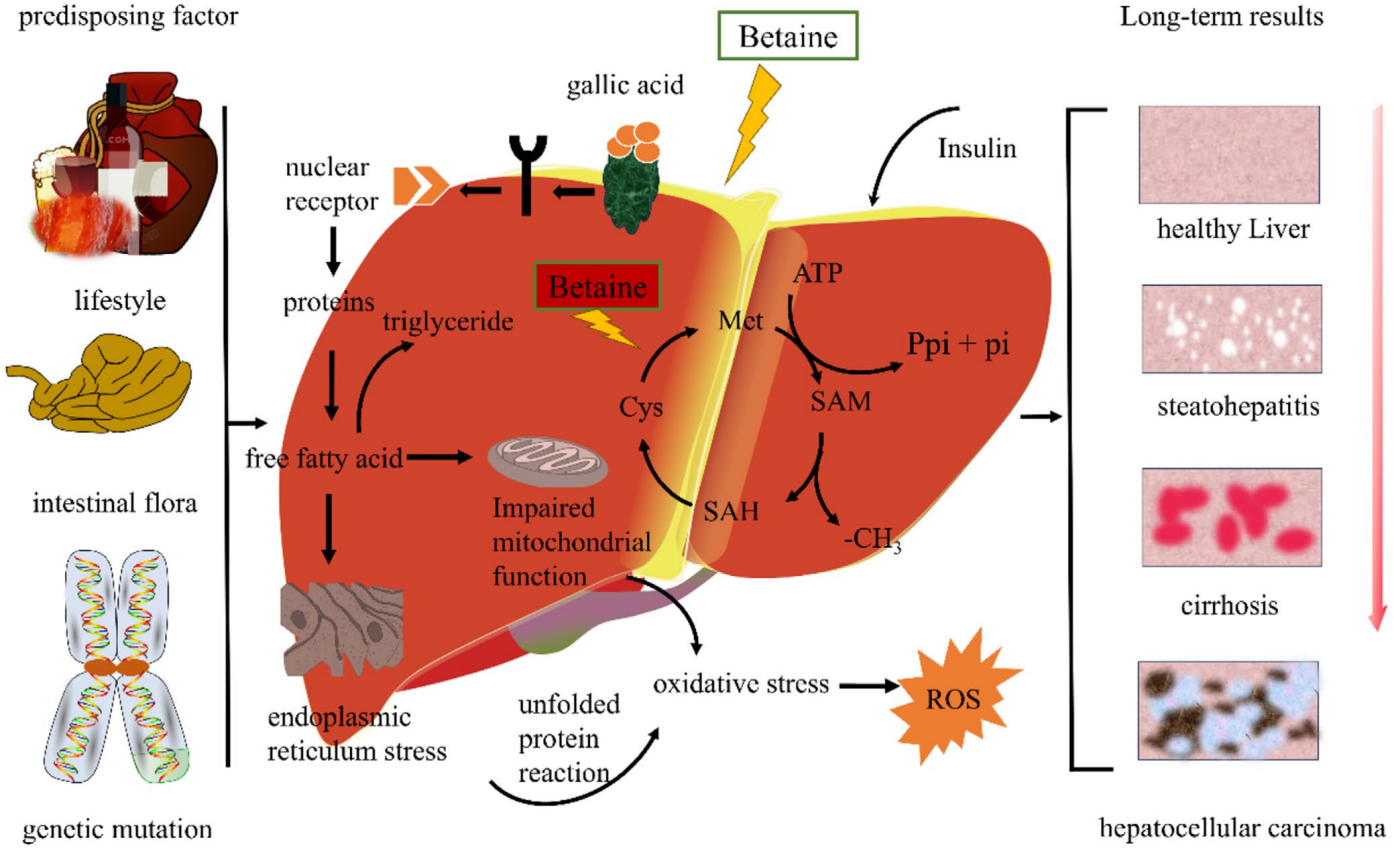
Effect of betaine on human health.

### 5.5 Antilipidemic and Antidiabetic Activity

Despite efforts to promote personal health, obesity rates continue to rise, threatening public health and economic development. Excess fat leads to metabolic issues, while lipase activity is key to lipid absorption and preventing related conditions. It has been demonstrated that betaine supplementation mitigates the adverse effects of a high‐fat diet (HFD) on gut microbiota by promoting the proliferation of anti‐obesity strains such as *akkermansia muciniphila* , *lactobacillus*, and *bifidobacterium*. The functional role of betaine in preventing high‐fat diet (HFD)‐induced obesity, metabolic syndrome, and the inactivation of brown adipose tissue was significantly enhanced in mice devoid of gut microbiota. *akkermansia muciniphila* serves as a crucial mediator for betaine by improving microbiome ecology and increasing the abundance of short‐chain fatty acid (SCFA)‐producing strains. The elevation of two principal SCFAs—acetate and butyrate‐significantly influences DNA methylation levels at the host miR‐378a promoter, thereby impeding the onset of obesity and glucose intolerance; however, these effects were partially counteracted by YY1, a common target gene within the miR‐378a family (Figure 3). Thus, it can be concluded that betaine ameliorates obesity and related metabolic syndrome through the gut microbiota‐derived miR‐378a/YY1 regulatory axis, revealing a novel mechanism whereby gut microbiota enhances host health (Du et al. 2021). Inorganic arsenic has been shown to increase incidences of poor glucose tolerance and diabetes mellitus. Additionally, betaine plays an essential role in homocysteine metabolism as a methyl donor. In their study, Esfahani et al. (2023) administered distilled water alongside doses of 500 mg/kg betaine or 10 mg kg^−1^ NaAsO_2_ to mice after a 28‐day period; on day 29, they fasted overnight before measuring fasting blood glucose levels followed by conducting a glucose tolerance test to assess betaine's effect on sodium arsenite‐induced diabetes and hepatotoxicity in mice. On day 30 post‐treatment initiation, mice were anesthetized for blood sample collection from cardiac sites while serum factors—including glutamate aminotransferase (ALT), aspartate aminotransferase (AST), alkaline phosphatase activity, and oxidative stress markers such as malondialdehyde along with glutathione levels were measured alongside superoxide dismutase, glutathione peroxidase, and catalase activities. Additionally, a comprehensive histopathological examination was performed on liver tissues. The findings indicate that arsenic exposure results in glucose intolerance coupled with oxidative/inflammatory liver injury; conversely, betaine administration effectively prevented arsenic‐induced hepatotoxicity along with mitigating glucose intolerance among treated subjects. The administration regimen utilizing a 500 mg/kg dose exhibited superior outcomes compared against other dosages applied. In light thereof, it may be inferred that betaine could serve potential therapeutic applications targeting diabetogenic/hepatotoxic repercussions stemming from arsenic exposure. Furthermore, a separate investigation revealed dietary betaine normalizes both glycemic/lipid homeostasis, thereby reducing incidence rates associated with obesity. A high‐calorie energy diet induced weight gain among murine models subsequently receiving daily gavage treatment comprising120mg kg^−1^ body weight over 4 weeks. Histological, biochemical, and hormonal analyses confirmed reversal regarding elevations observed across multiple microRNA species, including miR‐27a, miR‐27b, miR‐34a, miR‐200b, and miR‐223, within obese cohorts (Jin et al. 2021). In conclusion, betaine emerges as a viable candidate therapeutic agent addressing antidiabetic/hepatoprotective concerns anticipating clinical applicability.

### 5.6 Hepatoprotective and Radioprotective Activity

Betaine is primarily acquired through dietary sources and functions as an Osmo protectant in the kidney, while its principal role in the liver is to act as a methyl donor involved in metabolic processes (Figure 4). In both the liver and kidney, a specific betaine transporter protein facilitates the cellular uptake of betaine from plasma. In the kidney, betaine is taken up from the blood via the Na^+^ and Cl^−^ dependent betaine‐GABA transporter protein (BGT1) on the basolateral plasma membrane. Intracellular betaine, in conjunction with other organic molecules that regulate osmotic pressure, serves to balance high extracellular osmotic pressure and maintain normal cell volume. Furthermore, accumulation of betaine up to a certain concentration does not result in cellular damage (Neuhofer and Beck 2005). In the liver, betaine primarily functions to reinforce stability during DNA synthesis and repair. Day et al. (Day and Kempson 2016) discovered that cardiovascular disease, neurotransmitter imbalance, cancer, diabetes, aberrant immune function, and chronic inflammation are linked to diminished or disorganized methyl donors (Figure 3). In models of acute and chronic liver injury, including bile duct ligation (BDL) as a model of chronic liver injury and thioacetamide (TAA)‐induced hepatotoxicity as a model of acute liver injury. Furthermore, an increase in serum markers of liver tissue injury, as well as alterations in liver histopathology, were observed. The levels of tissue markers of oxidative stress were found to be significantly elevated in animals that had undergone bile duct ligation (BDL) and thioacetamide (TAA) treatment. Furthermore, hepatic mitochondrial function was found to deteriorate in both models. Betaine supplementation (10 and 50 mg kg^−1^, intraperitoneal injection) was observed to ameliorate liver injury by slowing down liver histopathological changes, significantly reducing tissue markers of oxidative stress, and attenuating serum biomarkers of hepatotoxicity. Conversely, betaine (10 and 50 mg kg^−1^, intraperitoneal injection) demonstrated protective effects on hepatocyte mitochondria. These findings suggest that the antioxidant and mitochondrial modulatory properties of betaine may be a key factor in its hepatoprotective mechanism (Heidari et al. 2018). Betaine modulates the phosphorylation of sulfur amino acid (SAA)‐related metabolites (e.g., S‐adenosylmethionine and homocysteine) as well as AMP‐dependent protein kinase (AMPK) and acetyl‐CoA carboxylase (ACC), thereby ameliorating fatty liver induced by a high‐fat diet (CDAHFD). CDAHFD‐induced endoplasmic reticulum stress (as indicated by BiP, ATF6, and CHOP) and apoptosis (as indicated by Bax, cleaved caspase‐3, and cleaved PARP) were reduced. To ascertain the role of autophagy in the alleviation of nonalcoholic fatty liver disease (NAFLD), the autophagy inhibitor chloroquine (CQ) was administered to mice fed a CDAHFD and betaine (0.5% in the drinking water). CQ did not impact SAA metabolism but diminished the beneficial effects of betaine, including increased hepatic lipids, endoplasmic reticulum stress, and apoptosis. It is noteworthy that the betaine‐induced improvement in lipid metabolism, as determined by protein levels of p‐AMPK, p‐ACC, PPARα, and ACS1, was reversed by CQ. Therefore, the activation of autophagy represents a crucial upstream mechanism through which betaine exerts its hepatoprotective effects, including the inhibition of steatosis, endoplasmic reticulum stress, and betaine‐ induced apoptosis (Seo et al. 2023). The potential role of oxidative stress, apoptosis, and fibrosis in radiohepatic injury remains unclear, despite their purported importance in this process. Shedid et al. (2019) irradiated mice in three doses at 3 Gy per week, resulting in a total dose of 9 Gy. Following the initial radiation dose, the rats were orally administered betaine (400 mg kg^−1^ d^−1^), and this supplementation continued throughout the irradiation period. The animals were euthanized 1 day following the final dose. It was demonstrated that a notable elevation in malondialdehyde, protein carbonyls, and 8‐hydroxy‐2‐deoxyguanosine, accompanied by a considerable decline in catalase activity and glutathione (GSH) content, resulted in oxidative stress within the liver following irradiation. The activity of the detoxifying enzyme cytochrome P450 (CYP450) was increased, while the activity of glutathione transferase (GSH‐T) was decreased. The increased activity of the apoptotic marker caspase‐3 was accompanied by elevations in hyaluronic acid, hydroxyproline, laminin (LN), and type IV collagen. These changes were associated with significant elevations in markers of liver dysfunction, including gamma‐glutamyl transpeptidase, alkaline phosphatase, and alanine and aspartate aminotransferases. Betaine treatment was observed to significantly attenuate oxidative stress, decrease CYP450 activity, enhance GSH‐T, and decrease caspase‐3 activity, as well as fibrosis marker levels, while significantly improving liver function. In conclusion, betaine exerts its beneficial effects on radiation‐induced liver injury and the progression of hepatic fibrosis by enhancing hepatic detoxification and reducing apoptosis, due to its antioxidant activity.

### 5.7 Antiviral Activity and Diuretic Effect

Betaine has possess antiviral and anti‐inflammatory properties. The administration of betaine to newly hatched commercial broilers via their drinking water for 3 weeks resulted in infection with the infectious bursal disease virus (IBDV). At 5 days post‐infection, bursal lesions were examined, and the mRNA expression levels of the IBDV VP2 gene, pro‐inflammatory cytokines, and interferon were quantified. Additionally, the levels of 5‐methylcytosine in the CpG sites within the promoter regions of IL‐6 and interferon regulatory factor 7 (IRF7) were assessed. Following IBDV infection, a notable increase in mRNA levels of VP2, IL‐6, type I (IFNα and IFNβ), type II (IFNγ) interferons, and IRF7 was observed alongside a reduction in follicular lymphocytes and decreased bursa inflammation. The levels of CpG island methylation in the promoter regions of IL‐6 and IRF7 significantly declined after IBDV infection. Administration of betaine was found to mitigate the severity of IBDV‐induced bursal lesions; furthermore, expression levels of IL‐6, IFNβ, and IRF7 mRNA were suppressed in response to IBDV presence. Betaine also appeared to counteract hypomethylation effects induced by IBDV on the promoter regions of both IL‐6 and IRF7 genes. These results indicated a correlation between IBDV‐induced expression levels of IL‐6 and IRF7 genes with inhibition of promoter region methylation. It is proposed that betaine supplementation may attenuate IBDV‐induced bursal injury through epigenetic regulation (Zhang et al. 2019). Hypertension is associated with electrolyte disturbances as well as end‐organ damage; conversely, betaine functions as an osmoregulator that protects cells from electrolyte imbalances and osmotic pressure—particularly within renal tissues. This study aimed to assess betaine levels across two distinct rat models: normotensive rats (Wistar‐Kyoto; WKY) versus spontaneously hypertensive rats (SHR), which serve as a model for hereditary hypertension. To achieve this objective, high‐performance liquid chromatography‐mass spectrometry (HPLC–MS) was employed along with real‐time PCR analysis coupled with protein blotting techniques assessing gene/protein expressions related specifically towards selected renal betaine transporter proteins SLC6a12/SLC6a20. The findings demonstrated markedly reduced serum, blood, lung, liver, and renal medulla concentrations among SHR compared against normotensive counterparts. Additionally, these changes correlated significantly regarding urinary excretion rates (0.20 ± 0.04 vs. 0.09 ± 0.02 mg^−1^ 24 h^−1^ 100 g^−1^ bw, *p* = 0.036). Administration regimen utilizing betaine enhanced diuretic efficacy without exerting significant impacts upon arterial blood pressures; the diuretic responses proved more pronounced among spontaneously hypertensive cohorts (SHR) than Wistar‐Kyoto (WKY) rats. Renal expressions pertaining towards aforementioned transporters remained statistically indistinguishable between groups. Increased renal excretions led directly towards reductions concerning protective osmotic agents' concentrations present within hypertensives' tissues. Results indicate potential causal relationships linking depletion states involving betaine alongside organ damages stemming from hypertension including those affecting kidneys. Furthermore, they suggest promising applications wherein betaine could function effectively serving roles akin to diuretics (Mogilnicka et al. 2024).

### 5.8 Human Health Promotion by Betaine

#### 5.8.1 Pathogen Prevention

Betaine exerts a significant effect on the prevention of intestinal inflammation, the reduction of inflammatory molecule expression, and the modulation of nuclear NF‐κB protein levels in colitis mouse models (Macias‐Ceja et al. 2016). Hepatitis B (HBV) is a globally prevalent pathogen responsible for hepatitis B. Yi and colleagues (Yi et al. 2021) developed a simple, visual, and rapid on‐site assay for HBV‐DNA based on a betaine‐assisted recombinase polymerase amplification followed by visual detection using lateral flow chromatography (BRPA‐LF) (Figure 3). The results indicated that when serum samples were subjected to recombinase polymerase amplification (RPA), non‐specific amplification occurred in the absence of purification. To enhance the stability of the RPA reaction, 0.8 M betaine was incorporated. The findings demonstrated that the BRPA‐LF method could detect 1000 copies of HBV‐DNA within a 50 μL mixture with sensitivities and specificities reaching up to 90% for serum samples. These results suggest that BRPA‐LF is resistant to serum interference and holds considerable potential for preventing HBV infection.

#### 5.8.2 Enhancement of Athletic Performance

There is a paucity of studies examining the biological activity of betaine in humans. However, its efficacy has been corroborated in animal models. The results obtained in animal models of newborn calves (Wang et al. 2019) and chickens (Song et al. 2021) were in accordance with the anticipated outcomes. These findings indicate that betaine has the potential to prevent cardiovascular disease and enhance physical performance. In instances where the body requires muscle relaxation following exercise, the administration of betaine may prove beneficial in reducing muscle damage and enhancing exercise capacity. To ascertain the efficacy of betaine in mitigating muscle damage following centrifugal exercise, male patients were administered varying doses of the compound after completing 100 jumps. Furthermore, betaine was administered as a supplement 24 and 48 h following the exercise session. The incidence of muscle pain was significantly reduced in subjects who ingested betaine compared to those who did not receive the supplement (Figure 3). A year later, the researchers conducted a similar study, comparing the effects of supplementing with beet juice versus consuming a nitrate‐rich beverage (Clifford et al. 2016). The results substantiated the substantial protective impact of betaine against muscle damage and demonstrated the efficacy of betaine in this regard. The protective effect of betanin was also evaluated following a marathon (Clifford et al. 2017). Professional runners ingested beet juice containing betaine for a period of 3 days following strenuous exercise. Furthermore, in addition to the rate of muscle damage as previously measured in other studies, blood tests were conducted to ascertain information regarding inflammatory markers, including blood levels of cytokines, white blood cells, creatine kinase (CK), C‐reactive protein (CRP) and aspartate aminotransferase. The measurements were taken both before and 2 days after the marathon. However, the results demonstrated that betaine did not exhibit a statistically significant reduction in muscle damage or inflammation relative to the control group who did not receive the betaine supplement. The researchers then postulated that this result may have occurred since the study subjects were professional athletes and that muscle damage was practically non‐existent after the marathon. It was therefore concluded that the ingestion of beet juice following a marathon was not protective against muscle damage, a finding that currently applies to professional athletes. In conclusion, there is no evidence to suggest that the ingestion of beet liquid containing betaine after a marathon is beneficial. It is recommended that future research be based on pre‐marathon treatment, as a brief, intense intake will not achieve the desired effect.

#### 5.8.3 Digestive System

Disturbances in the digestive system directly affect the physiological processes of food intake, digestion, and nutrient absorption. In a study conducted by Li et al. (2024) the effects of betaine on growth performance and intestinal health in rabbits fed varying levels of digestible energy were investigated. The findings indicated that rabbits receiving a low digestible energy (LDE) diet exhibited a significant reduction (*p* < 0.05) in body gain to feed intake ratio (G:F) and apparent total tract digestibility (ATTD). The ATTD was assessed over two periods: days 1–14 and days 1–36. Additionally, G:F was evaluated during these same intervals (days 1–14 and days 1–36), with results showing statistical significance (*p* < 0.05). The LDE diet was observed to up‐regulate the gene abundance levels of duodenal adhesion molecule 3 (JAM‐3) and down‐regulate the gene expression levels of ileum toll‐like receptor 4 (TLR4), myeloid differentiation factor 88 (MyD88), and interleukin‐6 (IL‐6) (*p* < 0.05). Additionally, amylase, lipase, trypsin activity, and immunoglobulin M levels were found to be reduced in the jejunum of the LDE‐treated group (*p* < 0.05). Furthermore, the addition of betaine to the LDE diet resulted in an increased ATTD of gross energy (GE), dry matter (DM), and organic matter in rabbits (*p* < 0.05). The gene abundance levels of ileal IL‐6 and duodenal JAM‐3 were observed to be upregulated (*p* < 0.05). In conclusion, the LDE diet was observed to decrease the activity of intestinal digestive enzymes and to lower the ATTD of nutrients. However, the addition of betaine to the LDE diet was found to improve the intestinal barrier structure and nutrient ATTD in rabbits, with the greatest effect observed when betaine was added at 500 mg kg^−1^. Moreover, betaine was observed to restore cortisol levels to normalcy in rats subjected to high‐salt stress (*p* < 0.05). The water intake and renal index of rats in the high‐salt stress group exhibited a statistically significant increase (*p* < 0.05). The aldosterone (ALD) levels were found to be decreased in all high‐salt diet groups (*p* < 0.05). Betaine supplementation was found to significantly reduce antidiuretic hormone (ADH) levels (*p* < 0.05). The administration of a high‐salt diet resulted in a reduction in the activities of amylase, lipase, trypsin, and pancreatic rennet within the lumen of the small intestine (*p* < 0.05). Conversely, the activities of these enzymes were observed to increase with betaine supplementation (*p* < 0.05). The height of the small intestinal villi was found to be significantly reduced in the group that had been fed a high‐salt diet (*p* < 0.05). However, the betaine content was found to be significantly higher in the betaine‐supplemented group than in the control group (*p* < 0.05). The administration of a high‐salt diet resulted in a reduction in the diversity of the intestinal flora, whereas the addition of betaine was observed to counteract these negative effects (Wang et al. 2018a).

#### 5.8.4 Lowering Blood Pressure

Cardiovascular disease is a significant contributing factor to hypertension. The body predominantly depends on dietary sources for betaine acquisition, and circulating betaine is primarily metabolized rather than renally excreted. In a study involving patients with a mean age of 52.6 ± 11.9 years (58.4% males), the mean interdialytic ambulatory systolic and diastolic blood pressures were 138.4 ± 22.7 mmHg and 84.4 ± 12.5 mmHg, respectively. The mean plasma betaine level was 37. The mean plasma betaine level was 6 μmol L^−1^, and multivariate linear regression analysis demonstrated a significant correlation between betaine and both systolic (*β* = −3.66, *p* = 0.003) and diastolic blood pressures (*β* = −2.00, *p* = 0.004). This association persisted even after adjustment for cardiovascular variables. However, betaine is metabolized differently in men and women, and this correlation was observed exclusively in female patients. Betaine cycling may prove an effective strategy for the management of blood pressure disorders in women (Wang et al. 2018b). Inhibition of angiotensin‐converting enzyme (ACE) activity has emerged as a primary therapeutic strategy for the management of hypertension (Hall et al. 2018). Betaine has been demonstrated to reduce hypertension by inhibiting ACE activity, with ACE inhibition ranging from 4.72% to 86.97% in undigested solid samples. All beet products demonstrated substantial potential for ACE inhibition, with ACE inhibitory activity values exhibiting a strong correlation with total betaine content (*r* = 0.836) and beet red content (*r* = 0.829) (Wang et al. 2018a). Additionally, betaine has been observed to induce arteriolar vasodilation, with 3 mM and 10 mM concentrations demonstrating this effect. Notably, the vasodilatory effect was significantly greater in intact vascularized rings compared to de‐endothelialized ones. However, exposure of coronary arteries to betaine did not influence the responses to two endothelium‐dependent vasodilators, specifically bradykinin (a receptor‐dependent agonist) and A23187 (a receptor‐independent agonist). The results indicate that although betaine, at physiological concentrations, does not significantly promote vasodilation or enhance vascular endothelial function in the short term, there is no evidence to suggest that betaine will not have a positive effect if administered over an extended period (Tawa et al. 2020). In conclusion, further verification is required to substantiate the therapeutic effect of betaine on blood pressure.

#### 5.8.5 Prevention of Alcoholic Liver Disease

Excessive alcohol consumption has been linked to a range of adverse health outcomes, including multiple organ damage, with the liver being a particular target organ. A reduction in the intrahepatic ratio of *s*‐Adenosyl‐l‐methionine (SAM) to s‐adenosylhomocysteine (SAH) has been observed in studies where chronic ethanol administration was the subject. This decreased ratio impairs the underlying methylation response, which in turn leads to alcoholic fatty liver (steatosis) and other features of alcohol‐related liver disease (ALD) (Dou et al. 2014). In contrast, betaine combination therapy has been demonstrated to normalize hepatocyte SAM:SAH ratios and to alleviate a few features associated with liver injury, including steatosis. The consumption of ethanol in the presence of early or concomitant betaine administration has been demonstrated to prevent or attenuate acute alcohol‐induced liver injury. Male C57Bl/6 mice were administered 6 g of ethanol per kilogram of body weight (BW) via gastric gavage on two consecutive occasions, with an interval of 12 h between administrations. Two independent groups of mice (*n* = 5 per group) were administered 4 g betaine per kg^−1^ BW via gavage 2 h before or simultaneously with ethanol or saline tube feeding. The mice were sacrificed 8 h after the final administration, and serum and liver parameters were quantified. The administration of ethanol in a binge‐like manner resulted in a 50% reduction in the hepatic SAM:SAH ratio and a more than threefold increase in hepatic triglycerides (*p* < 0.05). This latter change was accompanied by elevated serum glutamic oxaloacetic transaminase (AST) and glutamic pyruvic transaminase (ALT) activity and blood alcohol concentration (BAC), which was approximately three times higher than the legal limit for human intoxication. The administration of betaine prior to or concurrently with ethanol binge drinking resulted in a blood alcohol concentration (BAC) that was comparable to that observed in mice that received ethanol alone. Both betaine treatments led to a notable elevation in hepatic SAM levels in comparison to mice that were administered ethanol alone. This resulted in a normalization of the SAM:SAH ratio, a delay in the onset of hepatic steatosis, and the maintenance of other hepatic injury parameters (Arumugam et al. 2022). Li et al. (2011) observed a reduction in blood alcohol concentrations in rats fed an alcohol‐containing diet when provided with a diet rich in betaine. In a study where male volunteers consumed 375 mL of white wine for a month while another group took betaine and wine, this amino acid was found to mitigate the adverse effects of alcohol consumption by lowering homocysteine levels (Rajdl et al. 2016). Moreover, Shen et al. (2015) discovered that aberrant DNA methylation is linked to the development of ALD. This suggests that chronic alcohol consumption may result in DNA hypomethylation. Considering these findings, it can be posited that betaine, acting as a methyl donor, may play a pivotal role in the prevention of ALD (Figure 4).

## 6 Preservation Film

Betaine, an endogenous hormone, has been demonstrated to possess antioxidant and antimicrobial effects and has been employed in the domain of postharvest research on fruits and vegetables (Figure 3). In this context, it is also worth mentioning that betaine is also used to synthesize human‐ and environmentally‐friendly new forms of known herbicides for the protection of crops growing in fields (Pernak et al. 2016). It was demonstrated that the soaking of mushrooms in a betaine solution resulted in minimal weight loss and respiration rate, and the opening of the cap and browning were inhibited after 12 days of storage at a low temperature (2°C). Meanwhile, the treated samples exhibited high levels of polyphenols and ascorbic acid and demonstrated enhanced structural integrity of cell membranes. Betaine inhibited PPO activity and increased the activities of antioxidant enzymes, including SOD, POD, and CAT, during the storage period. The effect of the 2 mM betaine‐treated group exhibited significantly superior results compared to the other treatment groups (*p* < 0.05). This finding led to the conclusion that the concentration of betaine treatment was appropriate for prolonging the storage period of mushrooms (Wang et al. 2015). The 20 mM GB treatment of fresh jujube (*Zizyphus jujuba Mill. cv*. Dongzao) demonstrated that betaine effectively inhibited the expression of PG, Cx, PME, and *β*‐Glu genes, thereby maintaining the content of cell wall components. Concurrently, the treatment increased the activities of antioxidant enzymes (APX, CAT, SOD, POD) and non‐enzymatic antioxidants (AsA, GSH), which collectively reduced the accumulation of ROS. Furthermore, the activities of energy‐metabolizing enzymes (H^+^‐ATPase, Ca^2+^‐ATPase, SDH, and CCO) and the expression of related genes were markedly elevated, leading to the maintenance of high energy levels (ATP, ADP, AMP, and EC) (Figure 3). In conclusion, betaine enhances ATP biosynthesis by increasing energy metabolism, thereby providing essential energy for the antioxidant metabolism of jujube, which delays the softening of postharvest dates (Zhang et al. 2023). Furthermore, the application of 1 mM betaine resulted in a significant reduction in the chilling injury (CI) index of bell peppers during postharvest storage. The observed improvement in CI was accompanied by a decrease in cellular leakage, malondialdehyde (MDA) content, and lipid peroxidation levels. Additionally, betaine treatment enhanced gene expression and enzyme activities of peroxidase (POD), catalase (CAT), ascorbate peroxidase (APX), and glutathione reductase (GR) within bell peppers. Collectively, these findings indicate that betaine enhances cold tolerance in bell peppers by stimulating antioxidant gene expression and enzyme activities, thereby mitigating potential damage caused by CI (Wang et al. 2016).

In conclusion, betaine demonstrates the capacity to maintain postharvest quality across fruits and vegetables. Nevertheless, this study is relatively preliminary; future research should prioritize elucidating the mechanisms by which betaine preserves the quality of fruits and vegetables through genomic, transcriptomic, and metabolomic analyses.

## 7 Conclusion and Outlook

Betaine exhibits various biological activities and is an optimal natural pigment, known to reduce oxidative stress, endoplasmic reticulum stress, inflammation, and cancer development. Its protective role in the liver is linked to methionine metabolism regulation. This article reviews betaine's biological activities and applications; however, its underlying mechanisms are not fully understood. Betaine also shows anti‐anxiety, anti‐obesity, and anti‐cancer properties that warrant further investigation due to their therapeutic potential for a range of human diseases. Additionally, betaine has promising industrial applications such as solar cells, eco‐friendly aluminum coatings, cosmetic dyes, food preservation films, surfactants, and animal feed. Despite its wide‐ranging uses, challenges remain that require further research; clinical studies on betaine's health benefits have yet to be conducted. Moreover, betaine is weakly stabilized in food matrices and degrades under light and certain metal ions; thus, developing encapsulation technologies like spray drying or emulsions is essential. While animal models have explored its health benefits, limited clinical studies exist to clarify these mechanisms‐making it crucial to conduct trials involving humans. The technology for industrial synthesis of betaine remains underdeveloped; future researchers can approach this topic from multiple perspectives based on its structure. For instance, the effects of betaine on liver health remain insufficiently validated through clinical trials. While its anti‐inflammatory properties have yielded certain results in animal models, the underlying mechanisms of its impact on human health require further in‐depth investigation. Moreover, the industrial synthesis of betaine is still in its nascent stages, with most commercially available betaine being plant‐derived. The high cost associated with extraction poses a significant barrier to large‐scale industrial production. In the future, a deeper exploration of betaine's structural characteristics and chemical properties could facilitate the development of more efficient and advanced synthesis technologies. Currently, research largely relies on speculation from animal models rather than robust clinical trials; therefore, it's vital to investigate the impact of betaine on human health through rigorous clinical studies while exploring other potential protective effects comprehensively.

## Author Contributions

**Donglan Luo:** formal analysis (equal), writing – original draft (equal). **Xiaogang Wang:** formal analysis (equal), writing – review and editing (equal). **Sen Cao:** conceptualization (equal), visualization (equal). **Liangjie Ba:** project administration (equal), supervision (equal). **Jianye Chen:** investigation (equal), supervision (equal).

## Conflicts of Interest

The authors declare no conflicts of interest.

## Data Availability

No data was used for the research described in the article.
